# Five-year oncological outcome after a single fraction of accelerated partial breast irradiation in the elderly

**DOI:** 10.1186/s13014-019-1448-0

**Published:** 2019-12-21

**Authors:** Rémy Kinj, Marie-Eve Chand, Jocelyn Gal, Mathieu Gautier, Daniel Lam Cham Kee, Jean Michel Hannoun-Lévi

**Affiliations:** 1 Department of Radiation Oncology, University of Côte d’Azur, Fédération Claude Lalanne, Nice, France; 2 Biostatistic unit, Centre Antoine-Lacassagne, 06189 Nice, France

**Keywords:** Breast cancer, Elderly, Accelerated partial breast irradiation, Brachytherapy, Single fraction, Hypofractionated regimen

## Abstract

**Background:**

To update the clinical outcome of an elderly women cohort with early breast cancer who underwent accelerated partial breast irradiation (APBI) based on a post-operative single fraction of multicatheter interstitial high dose–rate brachytherapy (MIB).

**Material and methods:**

A single institution retrospective cohort study was performed focusing on elderly patients (≥ 65 years old) presenting a low-risk breast carcinoma treated by lumpectomy plus axillary evaluation followed by MIB APBI. A single fraction of 16 Gy was prescribed on the 100% isodose. Clinical outcome at 5 years was reported based on local relapse free survival (LRFS), specific survival (SS) and overall survival (OS). Late toxicity was evaluated. Cosmetic results were evaluated clinically by the physician.

**Results:**

Between January 2012 and August 2015, 48 women (51 lesions) were treated. Median age was 77.7 years (range: 65–92) with a median tumor size of 12 mm (range: 3–32). Five patients (pts) presented an axillary lymph node involvement (4 Nmic, 1 N1). Invasive ductal carcinoma was the most frequent histology type (86.3%). With a median follow–up of 64 months (range: 56–71), no local relapse occurred while 1 pt. developed an axillary relapse (2.1%). No Grade 3 or higher late toxicity was observed while 16 late toxicities occurred (G1: 14 events [87.5%) mainly G1 breast fibrosis). The rate of excellent cosmetic outcome was 76.4%.

**Conclusion:**

We confirmed the safety of the process and remained encouraging clinical outcome of a post-operative single fraction of MIB ABPI in the elderly. This approach leads to consider a very APBI as an attractive alternative to intra-operative radiation therapy while all the patients will be good candidates for APBI in regards to the post-operative pathological report.

## Background

After more than two decades of clinical research, accelerated partial breast irradiation (APBI) is considered as an efficient and safe adjuvant treatment for low-risk breast cancer [1, 2]. American Society of Radiation Oncology (ASTRO) and Groupe Européen de Curiethérapie of the European Society for Radiotherapy and Oncology (GEC-ESTRO) now consider that, in a well selected population, described as “suitable” (ASTRO) and “low-risk” (ESTRO), adjuvant APBI can be proposed as an alternative to standard or slightly hypofractionnated adjuvant schedule (4 to 6 treatment weeks) [3, 4].

A single fraction of adjuvant APBI using multicatheter interstitial brachytherapy (MIB) permits the maximal reduction of transportations and alleviates the treatment related constraints mainly for elderly patients with frequent comorbidities [5]. The single fraction schedule was challenged in retrospective and prospective studies and remained encouraging outcomes [6–9]. We already reported the early clinical outcome of a cohort of elderly women treated by a post-operative single fraction of very APBI (vAPBI) but a longer follow-up was needed to assess consistent results [9].

The aim of this short report was to update the clinical outcome after vAPBI in our previously published cohort of elderly patients presenting low-risk breast cancer.

## Material and methods

All material and methods sections were previously described in details [9].

### Patient selection

This is a single institution retrospective study including elderly patients presenting with low-risk breast cancer who underwent lumpectomy plus axillary evaluation followed by a single fraction of high-dose rate (HDR) MIB APBI. The patient cohort combined women enrolled in a prospective phase I/II trial (SiFEBI; Clinical.gov #NCT01727011, [7]) and elderly frail patients treated before the SiFEBI trial opening. Briefly, inclusion criteria were as follow: elderly women 65 years and older, histologically proven breast carcinoma with free surgical margins, negative axillary evaluation. Patients were excluded in case of: sarcoma or lymphoma histology or with metastatic dissemination.

### Treatments

#### Breast surgery

Patients underwent lumpectomy with axillary management (sentinel lymph node or axillary dissection). Four to five clips were clamped by the surgeon to mark the tumor bed before closing the lumpectomy cavity [10].

#### Brachytherapy

Brachytherapy was performed according to the GEC-ESTRO Breast Cancer Working Group recommendations for MIB APBI [11]. A post-implant CT scan was performed in order to delineate the clinical target volume (CTV) based on clips, surgical cavity and pathological margins including a total safety margin of about 2 cm [12]. A single fraction of 16 Gy was prescribed to the 100% isodose. Dose constraints were as follow: D90% ≥ 105% of the prescribed dose, D100% ≥ 75%, V100 > 95% of the CTV, V150 ≤ 40%, V200 ≤ 15%; dose non-homogeneity ratio (DNR) ≤ 35% [7].

#### Systemic therapy

Systemic therapies such as adjuvant chemotherapy and/or hormonal treatments were dispensed according to the protocols used in the Antoine Lacassagne Cancer Center.

### Follow up

The radiation oncologist performed iterative monthly post-brachytherapy clinics during 3 months (acute brachytherapy side effects). Then, clinical surveillance was performed twice a year, alternatively with the surgeon, with a yearly mammogram. Late toxicities were evaluated by Common Terminology Criteria for Adverse Event v4 (CTCAE.V4.0). Cosmetic evaluation was performed according to Harvard criteria [13].

### Statistical analysis

Description of the study population and of the different investigated parameters was made using absolute and relative frequencies for the qualitative data and summarized using descriptive statistics such as median, extreme for quantitative data. Survival time was defined between the surgery date and the event date. Local relapse free-survival (LRFS), regional relapse free-survival (RRFS), specific (SS) and overall survivals (OS) were estimated using the Kaplan-Meier method. Patients still alive were censored at the date of last follow-up. Median follow-up with 95% confidence intervals was calculated by reverse Kaplan–Meier method. Data entry and data management were performed on Ennov clinical® system and were analyzed using R 3.2.2 for Windows®.

## Results

### Patient and tumor characteristics

Between January 2012 and August 2015, a total of 51 lesions from 48 patients (pts) were treated with a vAPBI. Patient, tumor and treatment features are reported in Table 1. Patient median age was 77.7 years [range: 65–92]. Most of patients were Performans Status (PS) 0 (85%). The most frequent location was the upper external quadrant (39.2%). Histological type was mainly invasive ductal carcinoma (86.2%). The median tumor size was 12 mm [range: 3–32] while, 4 pts. presented with a microscopic node involvement (Nmic) and 1 pt. was classified N1. The median surgical margin was 5 mm [range: 1–10]. One lesion was associated with peri-neural invasion. All the tumors but three had positive hormonal receptor status while Her-2 status was over-expressed in 5 pts (9.8%).

**Table 1:** Patient, lesion and treatment characteristics

Patient features	Number of patients	%/(min – max)
Median age (years)	77.7	65.2–92.3
Performans Status		
0	41	85.5
1	7	14.5
Tumor side		
Left	28	54.9
Right	23	45.1
Median tumor size (mm)	12	3–32
Tumor stage		
T1a	28	54.9
T1b	18	35.3
T1c	5	9.8
Axillary lymph node status		
N0	46	90.1
N1mic	4	7.9
N1	1	2.0
Histology type		
Invasive ductal carcinoma	44	86.3
Invasive lobular carcinoma	3	5.9
Other	4	7.8
Histological grade		
1	32	62.7
2	14	27.4
3	5	9.8
Hormonal status		
Positive	48	94.1
Negative	3	5.9
Her-2 status		
Over-expressed	5	9.8
Non-over-expressed	46	90.2
Peri-neural invasion		
Yes	1	1.9
No	50	98.1
Median Ki-67 (%) Median surg. Marg.(mm)	10 5	5–60 1–10
Implant procedure		
Intra operative	47	92.2
Post-operative	4	7.8
Median time interv. Surg./APBI (d)	7	1–63
Median number of lines	11	5–15
Median number of planes	2	1–3
Median CTV (cc)	44	11–124
Median V100% (%)	96	86–100
Median V150% (%)	34	23–48
Median V200% (%)	12	8–21
MedianDNR	0.35	0.23–0.56

*Median time interv. Surg./APBI* Median time between intervention and APBI Median surg. *marg.* median surgical margins, *DNR* Dose non-homogeneity ratio: V100/V150

### Treatment characteristics

The median time interval between surgery and vAPBI was 7 days [range: 1–63]. A median number of 11 vectors [range: 5–15] on 2 planes [range: 1–3] were implanted, mainly intra-operatively (92.2%). The median CTV was 44 cc [range: 11–124]. The median V100% was 96% [range: 86–100] (Table 1).

### Oncological outcome

**Fig. 1:**
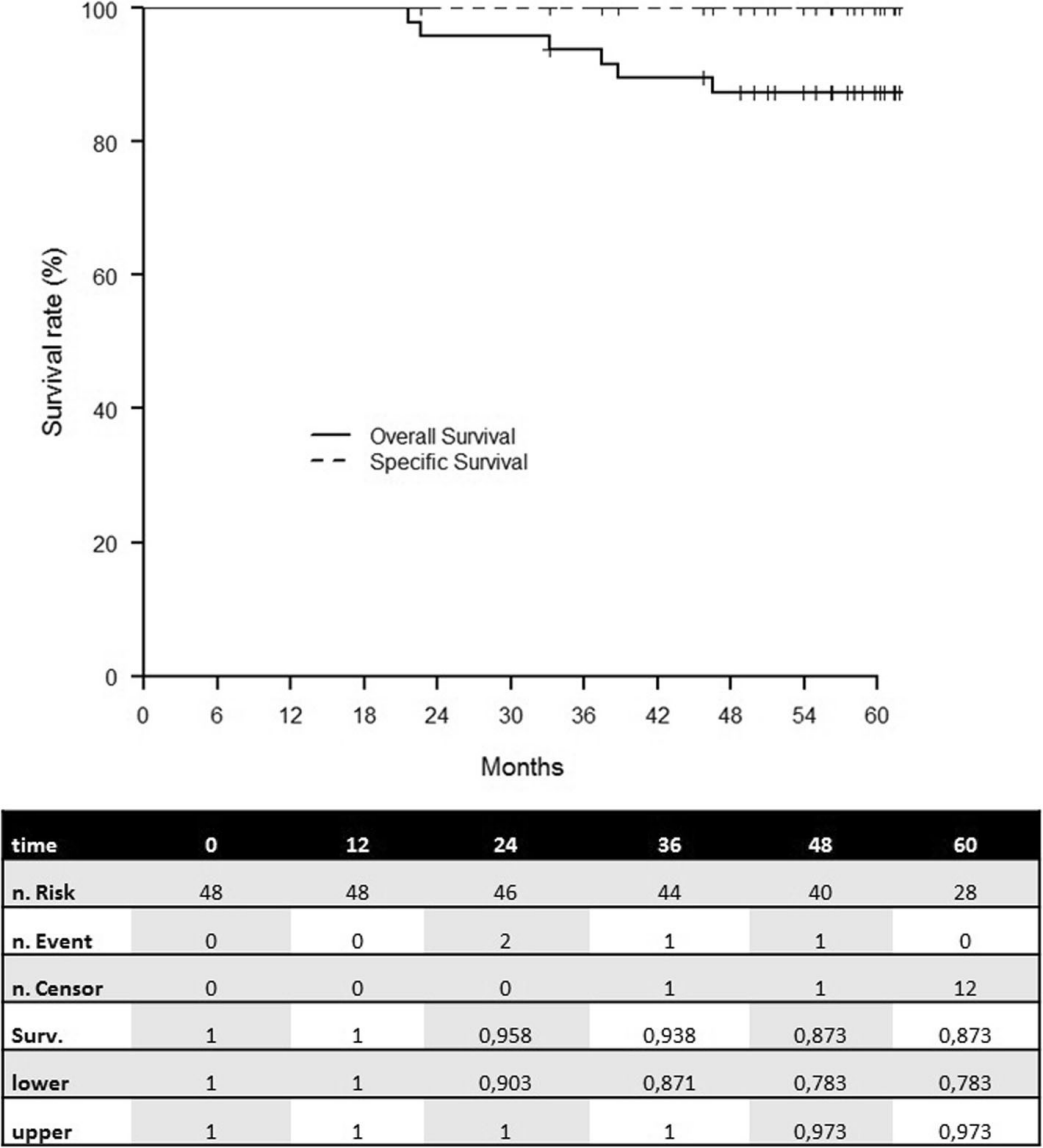
Overall and specific survival

With a median follow–up of 64 months [range: 56–71], no local relapse occurred while 1 pt. developed an axillary relapse (2.1%). Five-year LRFS, and SS were 100% and 5-year OS was 87.3% [78.3–97.3] (Fig. 1).

### Toxicity

Acute toxicity was previously reported [9]. No G ≥ 3 late toxicity was observed. Sixteen late toxicities occurred (G1: 14 events [87.5%]). G1 breast fibrosis and hypopigmentation of puncture site were the most frequent late side effects. The rates of excellent and good cosmetic outcome were 76.4 and 25.6% respectively. A breast asymmetry was noticed in 2 pts (4%) (Table 2).

**Table 2:** Late toxicity outcome

Late Toxicity	Number of events	%
Grade 1		
Breast fibrosis	5	31.3
Puncture site hypopig.	5	31.3
Telangectasia	2	12.5
Epithelitis	1	6.3
Other	1	6.3
Total	14	87.5
Grade 2		
Breast fibrosis	2	12.5
Total number of events	16	100
Cosmetic outcome	Number of estimations	%
Excellent	39	76.4
Good	12	25.6

*Puncture site hypopig* Puncture site hypopigmentation

## Discussion

This update short report confirms the excellent outcome of our cohort [9]. After a median follow-up of 64 months, no local relapse occurred while SS rate was 100% in this elderly cohort. The challenge in this population is alleviating the treatment, preserving oncological outcome and functional status. A vAPBI based on a post-operative single fraction appeared as an attractive technical option. Indeed, it drastically reduces the number of transportations, alleviates the treatment related constraints (mainly for elderly patients with frequent comorbidities) without compromise regarding local control [5]. Furthermore, the post-operative irradiation setting permits treating only validated candidates for APBI due to an appropriate definitive pathological report compatible with APBI criteria.

vAPBI also could be considered as a smart alternative to the omission of adjuvant radiation therapy which has been considered for elderly patients. Indeed, in phase III randomized trials which compared surgery + hormonal therapy with or without adjuvant WBI, there was a significant over-risk of local recurrence without breast irradiation [14, 15]. In our study, there was no local relapse and there was no cancer related death. Due to the deleterious impact of aromatase inhibitors on the quality of life [16], it is currently discussed to promote adjuvant breast irradiation without hormonal therapy in the elderly with low-risk breast cancer [17, 18].

Other studies reported encouraging results of vAPBI in patients presenting low-risk breast cancer and confirmed the safety and the efficiency of this new approach (Table 3) [6–8, 19–23].

**Table 3:** Very APBI discribed in the litterature

Authors	Year	# pts	MFU (months)	Irradiation techniques	Total dose (Gy)	D/f (Gy)	AG3 tox (%)	LG3 tox (%)	LF (%)	RF (%)	DM (%)	Ex/goodcosmetic results
Sacchini	2008	1834	31	HDR_IORT_	20/18	20/18	7.7	–	0	–	–	a
Khan	2013	30	11	Contura™	28	7 (BID)	0	0	–	–	–	–
Wilkinson	2012/17	45	74	Mammosite™	28	7 (BID)	13.3	2	0	0	0	91
Showalter	2016	28	6	HDR_IORT_	12.5	12.5	0	–	–	–	–	93
Latorre	2018	20	24	HDR_MIB_	18	18	0	0	0	0	5	80
Khan	2019	200	12	HDR_MIB_/Contura™	22,5	7,5	1.5	–	1	–	–	97
Jethwa	2019	73	14	Balloon	21	7	3	–	–	–	–	–
SiFEBI	2018/19	26	63	HDR_MIB_	16	16	7.6	0	0	0	–	88
Study	–	48	39	HDR_MIB_	16	16	6.3	0	0	2	0	100

*# pts.* number of patients, *MFU* Median follow-up, *HDR*_*IORT*_ High-dose rate brachytherapy performed intra-operatively, *MIB* Multicatheter interstitial high-dose rate brachytherapy, *Dose/f* Dose per fraction, *AG3tox* Acute Grade 3 toxicity, *LG3tox* Late Grade 3 toxicity, *LF* Local failure, *RF* Regional failure, *DM* Distant metastasis, *Ex/gd cosmetic results* Percentages of excellent and good cosmetic results, *APBI* Accelerated partial breast irradiation

^a^Cosmetic results were better with 18 Gy compared to 20 Gy

## Conclusion

We confirmed promising and encouraging clinical outcome of a post-operative single fraction of MIB ABPI in the elderly. This approach leads to consider vAPBI as an attractive alternative to intra-operative radiation therapy while all the patients will be good candidates for APBI in regards to the post-operative pathological report. VAPBI allows to drastically reducing the number of transportations and fatigue for elderly patients.

## Data Availability

The datasets used and/or analyzed during the current study are available from the corresponding author on reasonable request.

## Abbreviations

APBI: Accelerated partial breast irradiation
ASTRO: American Society of Radiation Oncology
DNR: Dose non-homogeneity ratio
GEC-ESTRO: Groupe Européen de Curiethérapie of the European Society for Radiotherapy and Oncology
HDR: High-dose rate
IORT: Intraoperative radiation therapy
LRFS: Local relapse-free survival
MIB: Multicatheter interstitial brachytherapy
OS: Overall survival
PS: Performans Status
RRFS: Regional relapse-free survival
SS: Specific survival
vAPBI: very APBI
WBI: Whole breast irradiation

## References

[1] Coles CE, Griffin CL, Kirby AM (2017). IMPORT Trialists. Partial-breast radiotherapy after breast conservation surgery for patients with early breast cancer (UK IMPORT LOW trial): 5-year results from a multicentre, randomised, controlled, phase 3, non-inferiority trial. Lancet.

[2] Strnad V, Ott OJ, Hildebrandt G (2016). Groupe Européen de Curiethérapie of European Society for Radiotherapy and Oncology (GEC-ESTRO). 5-year results of accelerated partial breast irradiation using sole interstitial multicatheter brachytherapy versus whole-breast irradiation with boost after breast-conserving surgery for low-risk invasive and in-situ carcinoma of the female breast: a randomised, phase 3, non-inferiority trial. Lancet.

[3] Correa C, Harris EE, Leonardi MC (2017). Accelerated partial breast irradiation: executive summary for the update of an ASTRO evidence-based consensus statement. Pract Radiat Oncol.

[4] Polgár C, Van Limbergen E, Pötter R (2010). GEC-ESTRO breast cancer working group. Patient selection for accelerated partial-breast irradiation (APBI) after breast-conserving surgery: recommendations of the Groupe Européen de Curiethérapie-European Society for Therapeutic Radiology and Oncology (GEC-ESTRO) breast cancer working group based on clinical evidence (2009). Radiother Oncol.

[5] Hannoun-Levi JM, Courdi A, Marsiglia H (2003). Breast cancer in elderly women: is partial breast irradiation a good alternative?. Breast Cancer Res Treat.

[6] Showalter SL, Petroni G, Trifiletti DM (2016). A novel form of breast intraoperative radiation therapy with CT-guided high-dose-rate brachytherapy: results of a prospective phase 1 clinical trial. Int J Radiat Oncol Biol Phys.

[7] Hannoun-Lévi JM, Lam Cham Kee D, Gal J, et al. Accelerated partial breast irradiation in the elderly: 5-Year results of the single fraction elderly breast irradiation (SiFEBI) phase I/II trial. Brachytherapy. 2019; in press. 10.1016/j.brachy.2019.10.007 31767533

[8] Latorre JA, Galdós P, Buznego LA (2018). Accelerated partial breast irradiation in a single 18 Gy fraction with high-dose-rate brachytherapy: preliminary results. J Contemp Brachytherapy.

[9] Kinj R, Chand ME, Gal J (2018). Single fraction of accelerated partial breast irradiation in the elderly: early clinical outcome. Radiat Oncol.

[10] Genebes C, Chand ME, Gal J (2014). Accelerated partial breast irradiation in the elderly: 5-year results of high-dose rate multi-catheter brachytherapy. Radiat Oncol.

[11] Strnad V, Major T, Polgar C (2018). ESTRO-ACROP guideline: interstitial multi-catheter breast brachytherapy as accelerated partial breast irradiation alone or as boost - GEC-ESTRO breast Cancer working group practical recommendations. Radiother Oncol.

[12] Strnad V, Hannoun-Levi JM, Guinot JL (2015). Working group breast Cancer of GEC-ESTRO. Recommendations from GEC ESTRO breast Cancer working group (I): target definition and target delineation for accelerated or boost partial breast irradiation using multicatheter interstitial brachytherapy after breast conserving closed cavity surgery. Radiother Oncol.

[13] Harris JR, Levene MB, Svensson G (1979). Analysis of cosmetic results following primary radiation therapy for stages I and II carcinoma of the breast. Int J Radiat Oncol Biol Phys.

[14] Hughes KS, Schnaper LA, Bellon JR (2013). Lumpectomy plus tamoxifen with or without irradiation in women age 70 years or older with early breast cancer: long-term follow-up of CALGB 9343. J Clin Oncol.

[15] Kunkler IH, Williams LJ, Jack WJ (2015). PRIME II investigators. Breast-conserving surgery with or without irradiation in women aged 65 years or older with early breast cancer (PRIME II): a randomized controlled trial. Lancet Oncol.

[16] Ferreira AR, Di Meglio A, Pistilli B, et al. Differential impact of endocrine therapy and chemotherapy on quality of life of breast cancer survivors: a prospective patient-reported outcomes analysis. Ann Oncol. 2019; In press. 10.1093/annonc/mdz298 31591636

[17] Buszek SM, Lin HY, Bedrosian I (2019). Lumpectomy plus hormone or radiation therapy alone for women aged 70 years or older with hormone receptor-positive early stage breast Cancer in the modern era: an analysis of the National Cancer Database. Int J Radiat Oncol Biol Phys.

[18] Ward MC, Vicini F, Chadha M (2019). Radiation therapy without hormone therapy for women age 70 or above with low-risk early breast Cancer: a microsimulation. Int J Radiat Oncol Biol Phys.

[19] Sacchini V, Beal K, Goldberg J (2008). Study of quadrant high-dose intraoperative radiation therapy for early-stage breast cancer. Br J Surg.

[20] Khan AJ, Vicini FA, Brown S (2013). Dosimetric feasibility and acute toxicity in a prospective trial of ultrashort-course accelerated partial breast irradiation (APBI) using a multi-lumen balloon brachytherapy device. Ann Surg Oncol.

[21] Wilkinson JB, Martinez AA, Chen PY (2012). Four-year results using balloon-based brachytherapy to deliver accelerated partial breast irradiation with a 2-day dose fractionation schedule. Brachytherapy..

[22] Khan AJ, Chen PY, Yashar C (2019). Three-fraction accelerated partial breast irradiation (APBI) delivered with brachytherapy applicators is feasible and safe: first results from the TRIUMPH-T trial. Int J Radiat Oncol Biol Phys.

[23] Jethwa KR, Park SS, Gonuguntla K (2019). Three-fraction Intracavitary accelerated partial breast brachytherapy: early provider and patient-reported outcomes of a novel regimen. Int J Radiat Oncol Biol Phys.

